# Genome of a giant isopod, *Bathynomus jamesi*, provides insights into body size evolution and adaptation to deep-sea environment

**DOI:** 10.1186/s12915-022-01302-6

**Published:** 2022-05-13

**Authors:** Jianbo Yuan, Xiaojun Zhang, Qi Kou, Yamin Sun, Chengzhang Liu, Shihao Li, Yang Yu, Chengsong Zhang, Songjun Jin, Jianhai Xiang, Xinzheng Li, Fuhua Li

**Affiliations:** 1 CAS and Shandong Province Key Laboratory of Experimental Marine Biology, Department of Marine Organism Taxonomy & Phylogeny, Center for Ocean Mega-Science, Institute of Oceanology, Chinese Academy of Sciences, Qingdao, 266071 China; 2 Laboratory for Marine Biology and Biotechnology, Qingdao National Laboratory for Marine Science and Technology, Qingdao, 266237 China; 3 Research Center for Functional Genomics and Biochip, Tianjin, 300457 China

**Keywords:** Giant isopod, Deep-sea, Genome assembly, Body size evolution, Oligotrophic adaptation

## Abstract

**Background:**

The deep-sea may be regarded as a hostile living environment, due to low temperature, high hydrostatic pressure, and limited food and light. Isopods, a species-rich group of crustaceans, are widely distributed across different environments including the deep sea and as such are a useful model for studying adaptation, migration, and speciation. Similar to other deep-sea organisms, giant isopods have larger body size than their shallow water relatives and have large stomachs and fat bodies presumably to store organic reserves. In order to shed light on the genetic basis of these large crustaceans adapting to the oligotrophic environment of deep-sea, the high-quality genome of a deep-sea giant isopod *Bathynomus jamesi* was sequenced and assembled.

**Results:**

*B. jamesi* has a large genome of 5.89 Gb, representing the largest sequenced crustacean genome to date. Its large genome size is mainly attributable to the remarkable proliferation of transposable elements (84%), which may enable high genome plasticity for adaptive evolution. Unlike its relatives with small body size, *B. jamesi* has expanded gene families related to pathways of thyroid and insulin hormone signaling that potentially contribute to its large body size. Transcriptomic analysis showed that some expanded gene families related to glycolysis and vesicular transport were specifically expressed in its digestive organs. In addition, comparative genomics and gene expression analyses in six tissues suggested that *B. jamesi* has inefficient lipid degradation, low basal metabolic rate, and bulk food storage, suggesting giant isopods adopt a more efficient mechanism of nutrient absorption, storage, and utilization to provide sustained energy supply for their large body size.

**Conclusions:**

Taken together, the giant isopod genome may provide a valuable resource for understanding body size evolution and adaptation mechanisms of macrobenthic organisms to deep-sea environments.

**Supplementary Information:**

The online version contains supplementary material available at 10.1186/s12915-022-01302-6.

## Background

The deep-sea environment is characterized by darkness, low temperature, high hydrostatic pressure, and lack of food. Despite this hostile environment, a growing number of deep-dwelling animals have been identified in this ecosystem, including worms, mollusks, fish, crustaceans, and so on [1, 2]. Crustaceans are one of the dominant invertebrates inhabiting deep-sea environment, and among them, some macrobenthos (e.g., giant isopods and amphipods) are specifically attractive as their body sizes are significantly larger than their shallow-water relatives [3]. Decoding genomes of these deep-sea species helps us understanding their unique adaptive mechanisms [4–8], whereas deep-sea crustaceans, including macrobenthos, lack relevant genome information.

**Fig. 1:**
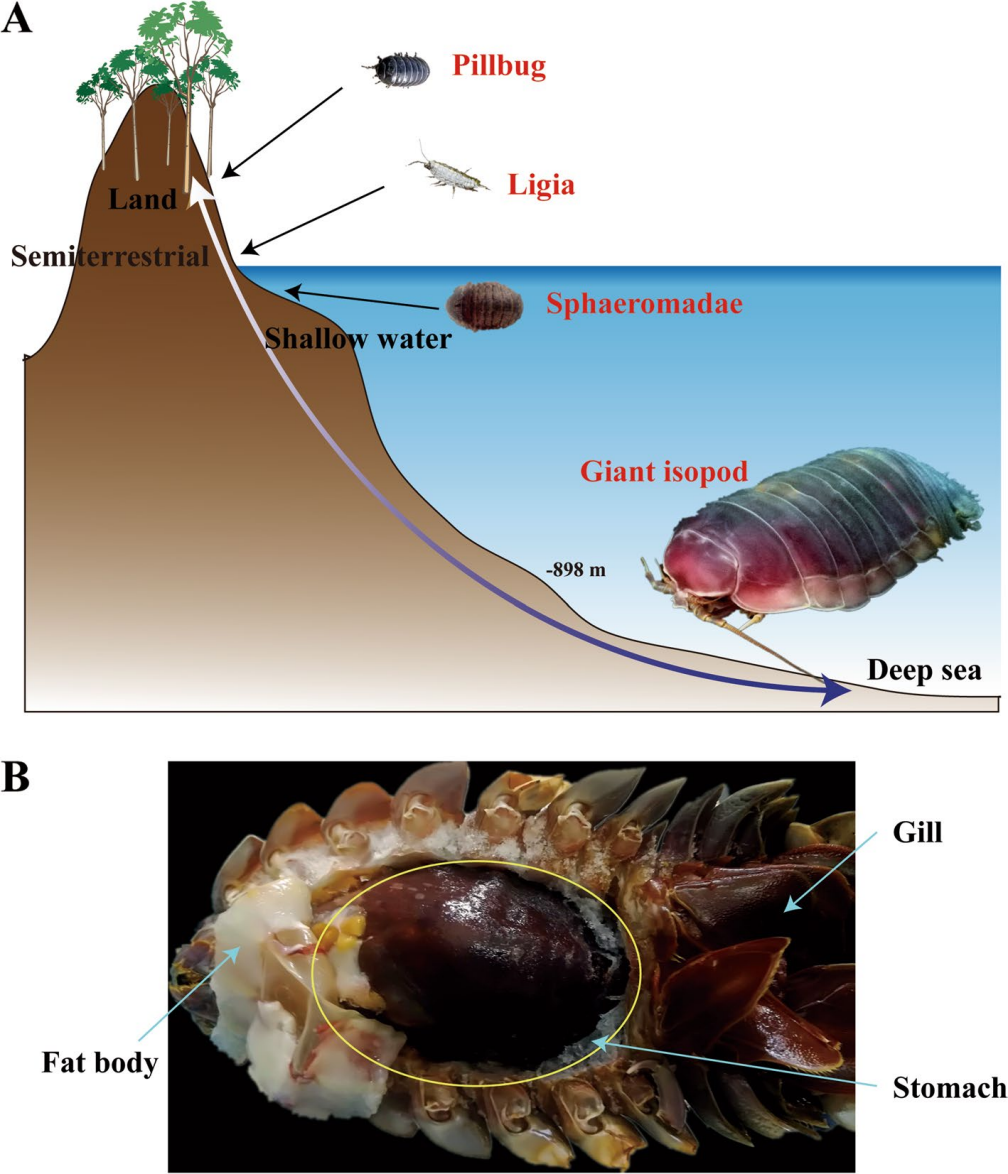
The distributions and phenotypes of isopods. **A** The distributions of various isopods from the land to deep-sea environments. **B** The morphology of the giant isopod, *B. jamesi*

Isopods are a large group of crustaceans with more than 10,000 species described. So far, Isopoda is one of the limited groups widely distributed in various environments, as they have been found in all oceans at different depths (from intertidal zone to hadal zone), in fresh and brackish waters, and on land (Fig. 1A) [9]. Therefore, Isopoda is an ideal model for studying migration and speciation, especially for the shift from shallow water to deep sea, and from ocean to land. Notably, isopods are one of the most morphologically diverse groups of crustaceans. Its size ranges from 0.5 cm (dwarf species) to as big as 50 cm for giant isopods [10]. Consistent with the Cope-Bergmann's Rule, isopods from deep sea tend to be larger than their relatives in shallower waters [11]. As the largest extant animals on the planet are aquatic and many of them are deep-sea organisms, the impact of marine habitats and evolutionary adaptation on body size is mysterious and noteworthy [12]. Besides, body size has always been regarded as one of the most important quantitative traits in evolutionary scrutiny, which is strongly correlated with many physiological and fitness characters [13]. Thus, isopods provide an excellent model for studying the adaptive evolution of body size, whereas, even with a great number of species, only two isopods, *Armadillidium vulgare* and *Armadillidium nasatum*, have been sequenced so far, and they are both terrestrial [14, 15]. Genomics of marine isopods, especially deep-sea species, is far from being understood.

Bathynomids (Crustacea: Isopoda: Cirolanidae) is regarded to be the “supergiant group” of isopods, which is well known for their big size [16, 17]. Bathynomids inhabit deep-sea benthic environment that are generally found on muddy bottoms at depths from 170 m to the dark of 2140 m [17, 18]. To adapt to the benthic environment, bathynomids adopt a burrowing behavior [19]. They have developed an oversized stomach, which can take approximately 2/3 of the whole body cavity when it fills up with food (Fig. 1B). In addition, midgut glands and adipocytes (collectively called “fat body”) are distributed throughout the body of bathynomids to store organic reserves [20]. Furthermore, bathynomids are well known for their extremely long fasting state (over 5 years), which should be the longest record to date [21]. Larger animals usually have greater absolute energy requirements [22]. However, the deep-sea conditions are harsh and food-limited, which seems to be unsuitable for the survival of giant animals [8, 23]. Therefore, a special efficient mechanism should be adopted by these supergiant isopods adapting to the deep-sea oligotrophic conditions.

Deep-sea expeditions provide excellent opportunities for us to uncover the unusual deep-sea creatures. During a recent expedition near Hainan Island in the northern South China Sea, a new deep-sea (a depth of 898 m) bathynomid species, *Bathynomus jamesi* Kou, Chen & Li, 2017, was collected and identified [24]. In this study, a high-quality genome assembly of *B. jamesi* was generated using PacBio sequencing technology. Analysis of the genomic characteristics identified potential factors related to the genome size evolution of *B. jamesi*. Based on the comparisons between the genomes of *B. jamesi* and its terrestrial relatives and other crustaceans, we have identified some expanded and positively selected gene families related to its body size evolution and deep-sea environment adaptation. This genome provides valuable resource for understanding evolutionary history of isopods and their deep-sea environmental adaptation mechanisms.

## Results

### Genome assembly and annotation

To estimate genome size of *B. jamesi*, a total of 235.25 Gb Illumina short reads were generated and utilized for genome survey analysis (Additional file 1 : Table S1). K-mer analysis indicated that the genome size of *B. jamesi* is approximately 5.24 Gb (Additional file 1: Fig. S1), which is larger than most crustacean genomes reported so far (generally < 2 Gb; Additional file 1: Table S2).

To assemble the genome of *B. jamesi*, 360.80 Gb PacBio long reads were generated and *de novo* assembled (Additional file 1: Table S1). The final assembly was 5.89 Gb in total length with a contig N50 length of 587.28 Kb, showing a higher continuity than the genomes of terrestrial isopod *A. vulgare* (contig N50 = 38.36 Kb) and many other crustaceans as well (Table 1) [14].

**Table 1:** Summary of genome assembly and characteristics of *B. jamesi* and other three crustaceans

Species	***B. jamesi***	***A. vulgare***	***L. vannamei***	***E. sinensis***
Genome size (bp)	5,892,409,081	1,725,108,002	1,618,026,442	1,562,256,418
Number of Contigs	22,827	52,740	50,304	12,722
Contig N50 (bp)	587,279	38,359	57,650	26,045
Contig N90 (bp)	108,712	18,318	14,641	2,670
Genome GC percent%	37.28%	29.15%	35.68%	46.39%
BUSCOs coverage (%)	94.80%	91.38%	94.00%	91.20%
Repeat percentage (%)	85.32%	69.54%	49.39%	45.30%
Gene number	23,221	19,051	25,572	28,033
Gene average length (bp)	936	1259	1546	1078
Exon number per gene	4.18	4.93	5.94	3.26
Exon average length (bp)	223	181	260	330
Intron average length (bp)	3010	1872	1484	1602

This genome assembly displayed a high quality as assessed by the coverage of raw Illumina sequencing data (99.80%) and RNA-seq data (84.23%) (Additional file 1: Table S3). Besides, a high proportion of BUSCOs (94.98%) were covered by the *B. jamesi* genome, which is comparable to or better than many recent sequenced crustacean genomes (Table 1; Additional file 1: Fig. S2, Table S4) [14, 25–27].

A total of 23,221 protein-coding genes were predicted and annotated in the *B. jamesi* genome (Table 1; Additional file 1: Fig. S3). The average intron length of genes (3010 bp) was significantly longer than that of *A. vulgare* (1872 bp) and many other crustacean genomes with relative smaller genome sizes (Table 1). It is consistent with the view that intron size is positively correlated with genome size [28].

### Repeats and genome size evolution

According to the Animal Genome Size Database (www.genomesize.com), C-value of isopods ranges from 1.71 to 8.82 pg, indicating there is a 5.2-fold variation of their genome sizes (Additional file 1: Table S5). *B. jamesi* has the largest genome (5.89 Gb) among sequenced crustacean genomes (Additional file 1: Table S2), which is about 3.4-fold larger than that of the *A. vulgare* genome (1.73 Gb) and approximately 49-fold of the clam shrimp *Eulimnadia texana* genome (0.12 Gb). Whole-genome duplication (WGD) has been identified to be one of the main factors causing genome expansion. However, only six syntenic blocks of paralogous genes were identified in the *B. jamesi* genome, which was far less than that of the horseshoe crab *Tachypleus tridentatus* (320 syntenic blocks), a species with WGD. In addition, Ks peak related to WGD and duplicated Hox gene cluster have not been identified in the *B. jamesi* genome (Additional file 1: Fig. S4). Therefore, *B. jamesi* appears to have not undergone WGD.

**Fig. 2:**
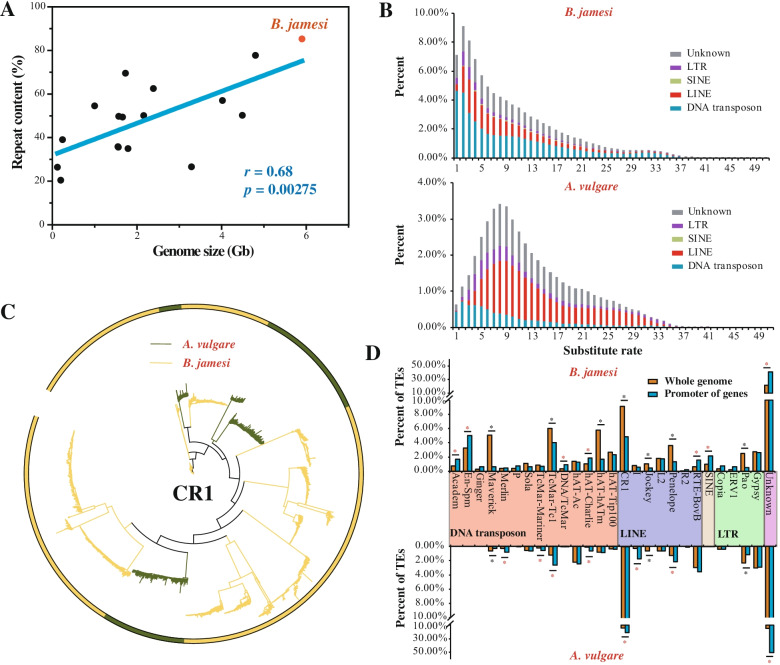
The evolution of transposable elements (TEs) and genome size. **A** The relationship between the genome size and repeat content. The repeat contents and genome sizes of the sequenced crustacean genomes were summarized in the Additional file 1: Table S2. The TE content and the genome size was positively correlated with the Pearson correlation *r* = 0.68 and *p*-value = 0.00275. **B** Kimura distance-based copy divergence analyses of TEs in the two isopod genomes, *B. jamesi* and *A. vulgare*. The graphs represent genome coverage for each TE superfamily in the different genomes analyzed. Clustering was performed according to their Kimura distances (*K*-value from 0 to 50). **C** Phylogenetic tree of the CR1 LINEs from *B. jamesi* (yellow) and *A. vulgare* (dark gray). **D** Enrichment analyses of TE families within gene promoters. The closest TE was calculated for each gene, and the content of the closest TEs were calculated and compared with that of the whole genome

K-mer analysis indicated that 89.7% of the *B. jamesi* genome was composed of repetitive sequences. Consistently, based on the RepBase and a local repeat database that generated by RepeatModeler, a total of 5.03 Gb sequences (85.32%) were annotated as repeats, which were significantly more than those of any other crustaceans (generally < 60%, *p* < 0.05) (Table 2; Additional file 1: Table S2). A strong positive correlation between repeat content and genome size has been identified among crustacean genomes (*r* = 0.68, *p* = 0.00275, Pearson’s test) (Fig. 2A), suggesting that repeat proliferation might be the major driving force for the genome expansion of *B. jamesi*.

**Table 2:** Comparison of the repeats among four crustaceans

Repeats	***B. jamesi***	***A. vulgare***	***L. vannamei***	***E. sinensis***
Total length	5.90 Gb	1.73 Gb	1.66 Gb	1.56 Gb
Repeats	85.32%	69.54%	49.39%	35.57%
DNA	35.99%	7.08%	9.33%	2.30%
DNA/En-Spm	3.28%	0.00%	6.39%	0.82%
DNA/Maverick	5.08%	0.63%	0.80%	0.10%
DNA/Merlin	0.37%	0.28%	0.00%	0.01%
DNA/TcMar-Mariner	0.87%	0.21%	0.06%	0.00%
DNA/TcMar-Tc1	6.05%	1.23%	0.03%	0.02%
DNA/hAT-Ac	1.41%	2.18%	0.00%	0.11%
DNA/hAT-Charlie	1.04%	0.11%	1.00%	0.09%
DNA/hAT-hATm	5.77%	0.81%	0.00%	0.00%
DNA/hAT-Tip100	2.67%	0.36%	0.00%	0.00%
LINE	19.36%	20.24%	2.82%	9.72%
LINE/CR1	9.13%	14.46%	0.25%	4.06%
LINE/Jockey	1.06%	0.63%	0.06%	0.05%
LINE/L2	1.80%	0.62%	0.35%	0.36%
LINE/Penelope	3.61%	1.26%	0.45%	0.04%
LINE/RTE-BovB	0.62%	3.00%	0.77%	0.91%
SINE	1.00%	0.00%	0.06%	0.29%
LTR	5.95%	5.89%	0.62%	1.79%
LTR/ERV1	0.24%	0.00%	0.02%	0.01%
LTR/Pao	2.48%	2.32%	0.00%	0.19%
LTR/Gypsy	2.76%	3.22%	0.22%	1.28%
Unknown	21.97%	14.87%	3.42%	10.39%
Satellite	0.31%	0.00%	0.10%	0.00%
Simple repeat	0.65%	18.08%	23.93%	6.90%
Low complexity	0.01%	3.57%	9.49%	2.04%

Transposable elements (TEs) and simple sequence repeats (SSRs) accounted for 84.27% and 0.65% of the *B. jamesi* genome, respectively (Table 2). Different from *B. jamesi*, the *A. vulgare* genome contained significant less TEs (47.89%) and more SSRs (18.08%) that similar with the SSR-rich species [25]. TEs accounted for 98.77% of the total repeats of *B. jamesi*, indicating their prominent role in genome expansion. DNA transposons (35.99%), long interspersed nuclear elements (LINEs, 19.36%) and long terminal repeats (LTRs, 5.95%) were three major groups of TEs in the *B. jamesi* genome (Table 2). The proportions of LINEs and LTRs in the genome of *B. jamesi* was similar to its terrestrial relative *A. vulgare*. Among them, two typical LINEs (CR1 and Penelope) and two types of LTRs (Pao and Gypsy) showed apparent proliferation in the genomes of *B. jamesi* and *A. vulgare*. In contrast to LINE and LTR, DNA transposon was the most abundant TE (35.99%) in the *B. jamesi* genome, and its content was significantly higher than that of *A. vulgare* (7.08%, *p* < 0.05). Five types of DNA transposons, including TcMar-Tc1 (6.05%), hAT-hATm (5.77%), Maverick (5.08%), En-Spm (3.28%), and hAT-Tip100 (2.67%), are significantly expanded in the *B. jamesi* genome in comparison with *A. vulgare* (*p* < 0.05, Table 2).

To assess the evolutionary history of TE proliferation, we performed a divergence time estimation of TEs. More than 95% of TEs have a divergence rate of < 20%, indicating that most TEs in the *B. jamesi* genome are relatively young (Fig. 2B). The CR1-type LINE was the most abundant TE of both *B. jamesi* and *A. vulgare*, which accounted for 9.13% and 14.46% of the two genomes, respectively (Table 2). However, phylogenetic analysis of the total CR1-type LINEs of the two genomes indicated these TEs proliferated independently in the two isopods, rather than derived from their ancestor (Fig. 2C). In contrast to *B. jamesi*, CR1-type LINEs were relatively more ancient in *A. vulgare* with a divergence rate of > 20% (Additional file 1: Fig. S5). As the most abundant TEs (2.12 Gb) in the *B. jamesi* genome, DNA transposons were also proliferated in a recent time like CR1 (Additional file 1: Fig. S5). Therefore, DNA transposons and CR1-type LINEs are the two major types of TEs that contribute to the genome expansion of *B. jamesi*, and this proliferation event should have occurred in a relative recent time.

TE proliferation can promote genome plasticity by altering genome structure or regulating gene expression. Previous studies suggested that TEs enriched in the promoters of genes play important roles in regulating gene expressions in response to different stresses [29]. Thus, we next analyzed the gene-surrounding TEs to investigate their potential functions. Different from the previous report that TEs are usually enriched in upstream and downstream of genes immediately (within 2 Kb) [29], TEs in the genome of *B. jamesi* were uniformly distributed surrounding genes (up- or down-stream of 10 Kb), especially for LINEs, LTRs and Maverick of DNA transposons (Additional file 1: Fig. S6). Exceptionally, TcMar, En-Spm, and hAT of DNA transposon and SINEs showed a relative slight enrichment surrounding genes (within 2 Kb). When analyzing the neighboring TEs of total genes, it was interesting to find that although many types of TEs (e.g., Maverick, TcMar-Tc1, hAT-hATm, CR1, Penelope, and Pao) proliferated significantly in the *B. jamesi* genome, they were less distributed surrounding genes than in other genomic regions (*p* < 0.05, Fig. 2D). In contrast, some TEs with lower abundance were significantly enriched in the promoters of genes, including Academ, En-Spm, TcMar-Tigger, hAT-Charlie, RTE-BovB, and SINE. Therefore, unlike the findings of the previous study [29], our results suggest the significant proliferation of TEs should perform a more profound impact on the plasticity of the whole genome than on the architecture of protein-coding genes in *B. jamesi*.

### Comparative genomics

**Fig. 3:**
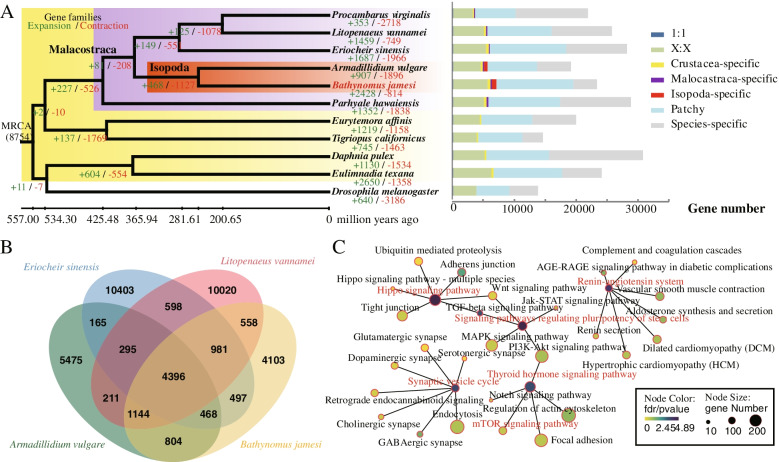
Comparative genomes analyses of *B. jamesi* and its relatives. **A** Phylogenetic tree and divergence times of *B. jamesi* and other arthropods. The number of significantly expanded (+, green) and contracted (−, red) gene families is designated on each branch. **B** Number of gene families shared among four Malacostraca species shown as a Venn diagram. **C** KEGG enrichment analysis of the expanded gene families of *B. jamesi*. The enrichment analysis was performed by using the toolkit from Omicshare (https://www.omicshare.com/). The enriched KEGG terms was referred to the Additional file 1: Fig. S7

Based on 177 orthologous single-copy genes, a phylogenetic tree was constructed to confirm the placement of *B. jamesi* (Fig. 3A). As expected, the two isopods (*B. jamesi* and *A. vulgare*) were clustered together and then nested by the other four malacostraceans. Isopods were estimated to be diverged from their last common ancestor around 366 million years ago (Mya), which is a time of the Late Devonian-Epoch. The deep-sea isopod (*B. jamesi*) and the terrestrial isopod (*A. vulgare*) were estimated to divergent around 245 Mya, which is consistent with the fossil records of Oniscidea (219.6–358.9 Mya) [30]. Besides, fossil record showing that another deep-sea isopod *Bathynomus giganteus* has emerged as early as 160 Mya [2]. Therefore, the deep-sea bathynomids should be originated between 160 and 245 Mya.

Comparative genomics analysis among 11 arthropod species identified 14,376 gene families. Among them, 418 gene families were isopod-specific (Fig. 3A), and 3683 gene families were specific in *B. jamesi* (Fig. 3B; Additional file 1: Table S6). Besides, a total of 274 significantly expanded gene families and 157 contracted families were identified in the *B. jamesi* genome (*p* < 0.05; Fig. 3A; Additional file 1: Table S7). These expanded gene families were functionally enriched in gene ontology (GO) terms related to membrane, peptidase activity, ion binding, proteolysis, and signal transduction (Additional file 1: Table S8). KEGG analysis significantly linked some of the expanded genes to Hippo signaling pathway, synaptic vesicle cycle, lipid metabolism (e.g., ether lipid metabolism and glycerophospholipid metabolism), and endocrine systems (e.g., renin-angiotensin system, insulin signaling pathway, and thyroid hormone signaling pathway) (Fig. 3C; Additional file 1: Fig. S7). The expansion of these gene families may reflect the adaptive evolution of *B. jamesi* to the deep-sea environment. Individual gene families related to body size evolution and deep-sea adaptation were discussed in greater depth in the later sections.

### Strengthened pathways related to large body size

*B. jamesi* is a giant isopod with a body length of > 20 cm, which is significantly larger than its intertidal and terrestrial relatives, e.g., sea roaches and pill bugs (generally < 3 cm). Comparative genomics approach helps us discover the genetic characteristics associated with the body size evolution of giant isopods.

**Fig. 4:**
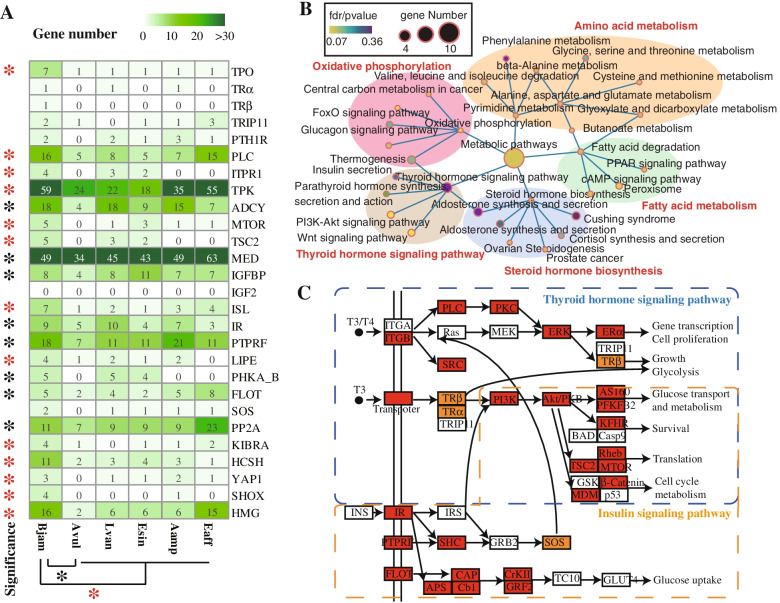
Gene expansion in growth-related hormone signaling pathways. **A** Heatmap of the number of genes involved in thyroid and insulin hormone signaling pathways among six crustaceans. The included species are *B. jamesi* (Bjam), *A. vulgare* (Avul), *A. amphitrite* (Aamp), *E. affinis* (Eaff), *L. vannamei* (Lvan), and *E. sinensis* (Esin). The red star indicates the correspondent gene of *B. jamesi* is significantly more than that of the other five crustaceans, and the black star indicates correspondent gene of *B. jamesi* is significantly more than that of *A. vulgare* but similar to other crustaceans (*p* < 0.05). **B** KEGG enrichment of the positively selected genes in *B. jamesi*. **C** The strengthened thyroid and insulin hormone signaling pathways. Genes with red background indicates significantly expanded genes of *B. jamesi* in comparison to *A. vulgare*; Genes with orange background indicates these genes are present in *B. jamesi* but absent in *A. vulgare*

As indicated from the above comparative genomic analysis, the expanded gene families of *B. jamesi* were significantly enriched in the thyroid hormone signaling pathway (*p* = 3.95E−06) (Fig. 3C; Additional file 1: Fig. S7), which is an important pathway in regulating growth, development and metabolism [31]. Many gene families related to thyroid hormone (TH) synthesis and secretion were significantly expanded and tandemly duplicated in the *B. jamesi* genome (Fig. 4A), including phosphatidylinositol phospholipase C (PLC), inositol 1,4,5-triphosphate receptor type 1 (ITPR1), tyrosine-protein kinase (TPK), adenylate cyclase (ADCY), serine/threonine-protein kinase mTOR (MTOR), tuberous sclerosis 2 (TSC2), and mediator of RNA polymerase II transcription subunit (MED). TH signaling is regarded as a key modulator of fundamental biological processes that has been evolutionarily conserved in both vertebrate and invertebrate species. Thyroid peroxidase (TPO), thyroid hormone receptor α (TRα) and β (TRβ), and thyroid receptor-interacting protein 11 (TRIP11) are four key enzymes in TH biosynthesis and signaling transduction. Seven TPO genes, one TRα gene, one TRβ gene, and two TRIP11 genes were identified in the *B. jamesi* genome, indicating the presence of endogenous TH in this deep-sea organism. In contrast, only a single gene encoding TPO and TRIP11 was identified in the *A. vulgare* genome, with the lack of TRα and TRβ genes. Likewise, the loss of TRα and/or TRβ genes has also been found in other crustaceans (Fig. 4A). In addition to the gene family expansion, the thyroid hormone signaling pathway has been identified to be under strong positive selection (six positively selected genes, KEGG enrichment *p* value = 9.93E−03) (Fig. 4B). Thus, in contrast to other crustaceans, *B. jamesi* has a complete thyroid hormone signaling pathway, which has been strengthened in the case that many genes of this pathway were significantly expanded and positively selected (Fig. 4C).

The insulin signaling is another hormone pathway that plays an important role in growth and development. In the *B. jamesi* genome, the insulin signaling pathway was also under significant enrichment of expanded gene families (*p* = 4.5E−02). A set of common genes involved in the insulin signaling pathway were identified to be tightly associated with body size evolution, including insulin growth factor (IGF), insulin-like growth factor-binding protein (IGFBP), insulin enhancer protein (ISL), and IGF receptor (IR) [32]. IGFs are normally bound to IGFBPs in great affinities that higher than IRs, and IGFBPs function as modulators of IGF availability and activity [33]. ISL is a LIM-homeodomain transcription factor involved in insulin secretion and metabolism, and also mediates glycolysis [34]. Like other crustaceans, IGF has not been identified in the *B. jamesi* genome, but IGFBP, ISL, and IR were all present in these crustaceans, indicating the presence of insulin signaling pathway. In contrast to *A. vulgare*, IGFBP complex acid labile subunit (IGFBP-ALS), ISL and IR were all significantly expanded in the *B. jamesi* genome (Fig. 4A). Apart from these genes, many other genes involved in this pathway were also expanded in *B. jamesi*, including receptor-type tyrosine-protein phosphatase F (PTPRF), hormone-sensitive lipase (LIPE), phosphorylase kinase alpha/beta subunit (PHKA_B), flotillin (FLOT), and MTOR (Fig. 4A). *MTOR* is the core gene of mTOR signaling pathway, which is also an important pathway in regulating animal growth and body size. This pathway locates at the downstream of the insulin signaling and thyroid hormone signaling pathway, and controls cell growth and metabolism in response to nutrients, growth factors, and cellular energy [35]. There are five genes encoding MTOR in the *B. jamesi* genome, which was significantly more than other crustaceans (Fig. 4A). In addition, there are four genes of the insulin signaling pathway (*INPP5B*, *PRKCI*, *PRKAG*, and *RHEB*) under positive selection in the *B. jamesi* genome (Fig. 4B). Therefore, similar to the thyroid hormone signaling pathway, the insulin signaling pathway of *B. jamesi* should also have been strengthened (Fig. 4C).

In addition to the two hormone signaling pathways, the Hippo signaling pathway was also significantly enriched by expanded gene families of *B. jamesi* (*p* = 3.44E−08)*,* which may make some contributions to the huge stomach and fat body of *B. jamesi*, because this pathway is functional important in controlling organ size [36]. Compared with *A. vulgare* and other crustaceans, many key genes in the Hippo signaling pathway, including dachsous (HCSH, 11 members), Protein Kibra (KIBRA, 4 members), transcriptional coactivator YAP1 (YAP1, 3 members), and serine/threonine-protein phosphatase 2A (PP2A, 11 members), were significantly expanded in the *B. jamesi* genome (Fig. 4A). Besides these genes, many other genes involved in body size were also expanded in *B. jamesi*, including short-stature homeobox protein (SHOX) and high mobility group protein (HMG) (Fig. 4A). The deficiency of these two genes (*SHOX* and *HMG*) would result in dwarfism [37]. Taken together, these strengthened growth-related signaling pathways may make great contribution to the large body size of *B. jamesi*.

### Molecular mechanisms underpinning deep-sea oligotrophic adaptation

To adapt to the deep-sea oligotrophic environments, the mechanisms of food storage and utilization of giant isopods should have undergone strong selective pressure. In accordance, giant isopods have developed a huge stomach to store food and can survive from an extraordinary long fasting state (> 5 years) (Fig. 1B) [21].

**Fig. 5:**
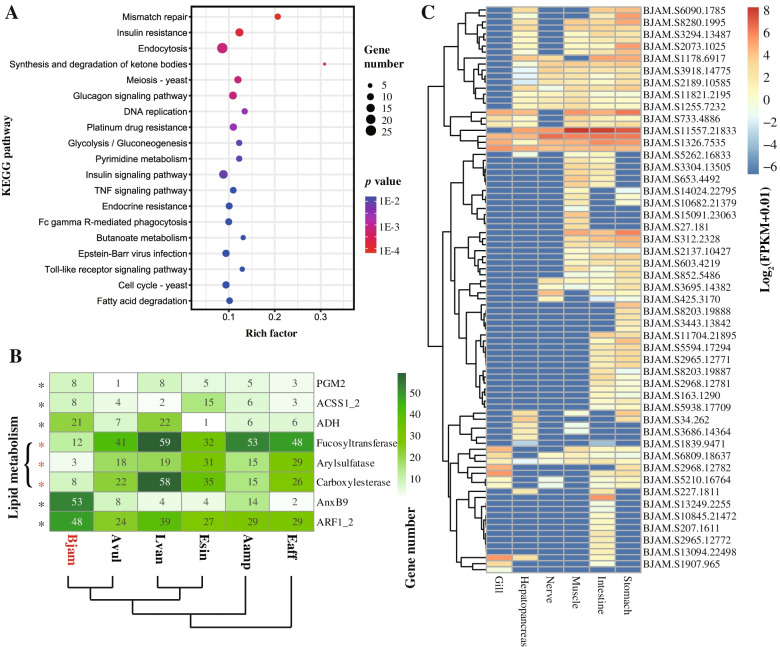
The differential gene expressions in six tissues of *B. jamesi*. **A** KEGG enrichment analysis of the highly expressed genes in stomach and intestine. The top 20 significantly enriched KEGG terms were displayed in the plot. **B** Heatmap of the number of genes involved in glycolysis, lipid metabolism and endocytosis among six crustaceans. The included species are *B. jamesi* (Bjam), *A. vulgare* (Avul), *A. amphitrite* (Aamp), *E. affinis* (Eaff), *L. vannamei* (Lvan), and *E. sinensis* (Esin). The red star indicates the correspondent gene of *B. jamesi* is significantly less than that of the other five crustaceans, and the black star indicates correspondent gene of *B. jamesi* is significantly more than that of the other five crustaceans (*p* < 0.05). **C** Expression level of the genes involved in the endocytosis of *B. jamesi*

In order to identify potential genes related to nutrient storage, absorption, and utilization, RNA-seq analysis was performed on six tissues of *B. jamesi*. A total of 901 genes were identified to be specifically highly expressed in digestive organs, including stomach and intestine. These differently expressed genes enriched in the pathways of mismatch repair, insulin signaling and resistance, endocytosis, glycolysis, and so on (Fig. 5A). Glycolysis is an important metabolic process in which glucose is broken down to produce energy. Genes involved in the glycolysis pathway were mostly highly expressed in the stomach, intestine, and muscle of *B. jamesi* (Additional file 1: Fig. S8). Among them, phosphoglucomutase-2 (PGM2) is a transferase that plays an important role in carbohydrate metabolism of both glycogenolysis and glyconeogenesis [38]. Eight genes encoding PGM2 were identified in the *B. jamesi* genome, whereas only one PGM2 gene was found in the *A. vulgare* genome (Fig. 5B). Besides, these genes were tandemly duplicated on the scaffold281 and scaffold7261 of the *B. jamesi* genome, and they were mostly high expressed in stomach and intestine. Similar results were also identified in the genes encoding acetyl-CoA synthetase (ACSS1_2) and alcohol dehydrogenase (ADH), both of which participate in the TCA cycle for ATP production. A total of eight *ACSS1_2*s and 21 *ADH*s were identified in the *B. jamesi* genome, which were significantly more than that of *A. vulgare* (four *ACSS1_2* and seven *ADH*s), and these genes were also highly expressed in the stomach and intestine. Therefore, *B. jamesi* may adopt an efficient mechanism of glycolysis to provide sufficient energy for its biological activities.

Since giant isopods developed fat body to store organic reserves [20], the lipid synthesis and metabolism may under natural selection. However, out of our expectation, none of gene families related to lipid or fatty acid synthesis showed any signatures of expansion or under positive selection. Instead, some gene families related to lipid degradation were significantly contracted in the *B. jamesi* genome, including genes encoding fucosyltransferase, arylsulfatase, and carboxylesterase (Fig. 5B). These proteins are supposed to function in degrading glycolipids, sphingolipid and many esters. In addition, two genes related to fatty acid degradation (*hcaD* and *echA*) were under positive selection (Fig. 4B). Therefore, the lipid accumulation in the fat body should result from low efficiency of lipid degradation rather than high efficiency of lipid synthesis.

**Fig. 6:**
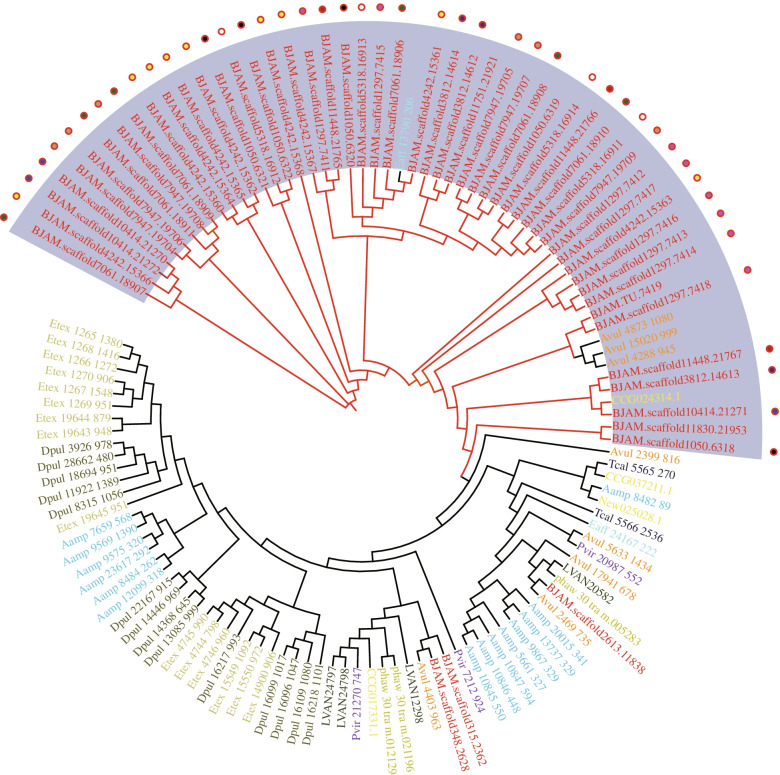
Phylogenetic tree of the genes encoding AnxB9. The AnxB9 genes from various crustaceans were used for the tree construction, which labeled in various colors. A cluster of AnxB9 genes was specific expanded in the *B. jamesi* genome (gray background), and these genes were tandem duplicated in the genome. The circles with different colors indicate the genes located on different scaffolds

Beside energy production, the molecule transportation is also important for the absorption and utilization of food. Vesicular transport is an important process of transporting macromolecules through membrane, which has been identified to be under strong natural selection in deep-sea crustaceans [39]. Endocytosis is an essential process of vesicular transport, which actively transports molecules into cell by engulfing it with its membrane. The pathway of endocytosis was significantly enriched by differentially expressed genes (*p* = 1.8E-03), and a large number of them were specifically expressed in the stomach and intestine (Fig. 5C). Besides, some expanded gene families were identified to be involved in vesicular transport, and annexin B9 (AnxB9) was a representative one among them. AnxB9 is a functional protein involved in the formation of multivesicular bodies and regulation of protein trafficking, and even in stabilizing the endomembrane system during stress [40]. A total of 53 genes encoding AnxB9 were identified in the *B. jamesi* genome, which were significantly higher than in *A. vulgare* (eight genes) and other crustaceans (seven genes on average). These AnxB9 genes were mostly tandem duplicated in the *B. jamesi* genome (Fig. 6), and some of them were highly expressed in stomach, intestine, and muscle. Therefore, the expansion of gene families and their specific expression in digestive organs play an important role in the energy supply of giant isopod and help these organisms adapt to the oligotrophic conditions of the deep-sea environment.

## Discussion

With the development of deep-sea diving and genome-sequencing technologies, a growing number of deep-sea organisms have been discovered, collected, and identified, and their genomes have been sequenced [4–8]. Genome sequencing sheds lights on variable adaptive mechanisms of these species to the deep-sea environment. However, no deep-sea crustacean genome has been reported so far even though Crustacea comprises one of the dominant groups of deep-sea organisms. In this study, we reported the first deep-sea crustacean genome and analyzed its genome characteristics, which provides a valuable resource for studying the unique mechanisms by which crustaceans adapt to deep-sea ecosystems.

A large genome size is one of the most apparent characteristics shared by the genomes of *B. jamesi* and some other deep-sea species [5, 6]. Previous studies suggested that genome size tend to be larger in deeper conditions as driven by low temperature and high hydrostatic pressures from deep-sea environment [3, 41]. Indeed, besides *B. jamesi*, large genome size has also been detected in some other deep-sea crustaceans (e.g., amphipod *Ampelisca macrocephola*: ~ 63 Gb) and polar crustaceans (e.g., Antarctic krill *Euphausia superba*: ~ 42 Gb) [3, 42, 43]. However, this rule seems inapplicable for isopods because some shallow-water species (e.g., *Sphaeroma destructor*, 6.79 pg), terrestrial species (e.g., *Oniscus asellus*, 8.60 pg) and parasitic species (e.g., *Nerocila munda*, 8.82 pg) also have large genome sizes (Additional file 1: Table S5). Even among sympatric pairs of deep-sea species there is a large variation in their genome sizes, suggesting an absence of a dominant environment variable influencing genome size [3, 41]. However, as summarized in previous studies [44, 45], a rough generalization indicated that marine and terrestrial isopods have larger genomes overall versus freshwater species, and groundwater isopods have larger genomes than their surface-water relatives. Thus, it is likely that larger genomes are specific to certain families or genera or groups that inhabit similar conditions, e.g., deep-sea bathynomids. However, more evidences of the genome size studies, especially on the deep-sea taxa, is needed to support our hypothesis.

The selective pressure from deep-sea may be a candidate factor in shaping the large genomes of bathynomids. In addition to deep-sea habitat, there are many other factors showed possible relationships with genome size, including body size and life-history strategy [3, 41, 45]. Indeed, a relationship between genome size and body size has been identified in amphipods, but it is limited in giant organisms [3]. Thus, bathynomids may also apply a similar rule with giant amphipods because it is also a group of organisms displays size gigantism. Besides, the life-history strategy, including low basal metabolic rate (BMR), low mobility, and high longevity, are presumed to be positively correlated with genome size [44]. As expected, these traits have been commonly identified in deep-sea bathynomids [46]. Thus, the large genome size of bathynomids may tightly associate with their ecological habitats, body sizes and life-history strategies. Furthermore, the body size and life-history strategy of bathynomids also displayed close relationships with their deep-sea habitats. Firstly, their body sizes showed a positive correlation relationship with the depths of their habitats (Additional file 1: Fig. S9). According to the Bergmann’s rule, organisms inhabiting higher latitudes tend to have larger body sizes; this would, in turn, also be applicable to the deep-sea organisms [3, 47]. Indeed, many deep-sea crustaceans, including giant squids, giant sea spiders, giant isopods, and amphipods, are significantly larger than their shallow-water relatives [3]. Thus, the large body size of these crustaceans may be tightly associated with their deep-sea habitats. Besides, the low BMR is a well-known characteristic of gigantic animals living in places with limited feeding resources, such as the deep-sea oligotrophic environment [48]. Therefore, the factors driving genome size evolution of bathynomids appear to be complex, which may be the result of a combination of deep-sea habitat, body size and life-history strategy.

The deep-sea environmental stress can disrupt the epigenetic control of TEs leading to TE proliferation and increased genome size [49]. TE proliferation has long been considered to be the major cause of the large genome size of many species, which is also a consequence of the genome of *B. jamesi* and some other deep-sea species (e.g., the deep-sea clam *Archivesica marissinica* and tubeworm *Paraescarpia echinospica*) [5, 6]. Notably, *B. jamesi* has the highest content of TEs (84.27%) among sequenced crustacean genomes, and it is also significantly higher than that of *A. marissinica* (55.10%) and *P. echinospica* (42.20%), making its genome also larger than these two deep-sea species (1.52 Gb and 1.09 Gb, respectively). Therefore, in contrast to these two species, TEs of *B. jamesi* should be more active during the evolutionary history. Similar to the genomes of *A. marissinica* and *P. echinospica*, DNA transposons and LINEs were also the two major components of TEs in the *B. jamesi* genome, suggesting these TEs are adaptively selected and highly dynamic in deep-sea genomes. In consistent, our results suggested DNA transposons and LINEs were intensively proliferated in a relative recent time in the *B. jamesi* genome. The large amount of DNA insertion or deletion would result in a high genome plasticity [50]. As a consequence, the proliferation of TEs has profound effects on genome size, structure, stability and plasticity, and finally contributes to adaptive evolution [41, 51]. Therefore, the convergent proliferation of DNA transposons and LINEs in deep-sea species might play an important role in shaping highly plastic genomes and helping them adapt to the deep-sea environment. In addition, the highly dynamics of TEs may be beneficial for *B. jamesi* adapting deep-sea environment better.

The enormous size of giant isopods is a classic example of a phenomenon known as deep-sea gigantism. By analyzing the genome of *B. jamesi*, we get a glimpse of the molecular basis of its giant size. The growth-related hormone signaling pathways, including thyroid and insulin signaling pathways, play important roles in regulating growth and body size. Thyroid hormone influences growth in part by altering the secretion and effects of growth hormone. Growth hormone, in turn, mediates its effects by regulating the synthesis and secretion of insulin-like growth factor-I (IGF-I) [52]. The cooperation of these hormone pathways can cause gigantism when excess growth hormone leads to increased linear growth [53]. In this study, strengthened thyroid and insulin hormone signaling pathways were identified in the *B. jamesi* genome, which might functionally contribute to its gigantism. The mTOR pathway is the primary cell-autonomous nutrient sensor, while circulating hormones such as IGF-I and thyroid hormone are the main systemic regulators of growth and maturation in animals [31, 34, 35]. In addition, proper organ growth is also monitored and coordinated with whole-body growth through modulation of Hippo signaling [36]. Many genes participating in these pathways have been expanded in the *B. jamesi* genome, resulting for a strengthened network of growth-related pathways. The large body size of *B. jamesi* could be explained by the cooperation of these strengthened pathways.

According to previous studies, the body size has been identified to be strongly correlated with the BMR, duration of juvenile growth and longevity [12]. In consistent, our anatomic result showed that *B. jamesi* has a huge stomach to store food such that the fulfilled stomach occupied about 2/3 of the body. It also has a large amount of fat body spreading all over the body cavity to store organic reserves. Besides, it is likely that *B. jamesi* also has an extraordinary long fasting state as the starvation record (> 5 years) is kept by the congeneric species *B. giganteus* [21]. Furthermore, the low BMR, slow growth, high longevity have been observed in giant isopods, which may be a strategy for the survival of these large organisms [46]. Therefore, in correlation with large body size, *B. jamesi* has bulk food storage, low BMR, slower growth, and greater longevity.

Generally, organisms with large body sizes have greater absolute energy requirements [22], whereas food is definitely a limiting factor in the deep sea, for less food being available in deeper water and at greater distances from shore, which seems unsuitable for the survival of large animals. Thus, there is a debate about the adaptive evolution of deep-sea large animals and especially its possible causes. Large-sized body would be beneficial for competition and predation, whereas more energy will be costed to sustain the large-sized body. Kleiber’s Rule states that “larger animals are more efficient” [54, 55]. In the deep sea, the smaller surface area to volume ratio gives the giant isopods the advantage of conserving greater energy, with less energy lost to the surroundings through heat. Yet, the disadvantage of gigantism is that the absolute energy demand is large. It will be much more difficult to obtain adequate energy in the deep-sea environment, where food is usually an extremely scarce resource. To achieve a balance, it is likely that *B. jamesi* has adopted a sequence of survival strategies including low BMR, specialized energy storage organs, and efficient nutrient absorption and utilization. In this study, our results suggest *B. jamesi* have developed an efficient pathway for glycolysis and vesicular transport, which will contribute to its nutrient absorption and utilization. In addition, *B. jamesi* has low efficient of lipid degradation to support its lipid accumulation in fat body. Therefore, in contrast to small animals, an efficient mechanism of nutrient storage, absorption and utilization could be more important for the macrobenthos to adapt the deep-sea oligotrophic environment.

## Conclusions

The genome of a deep-sea giant isopod *B. jamesi* was successfully assembled, representing the first high-quality genome of deep-sea crustaceans. Comparative genomic analyses provided new insights into the evolution of genome size and body size of animals and the adaptive mechanisms to the deep-sea extreme environments. The isopod genomes will shed lights on the habitat shift and evolution history of the crustaceans inhabiting deep-sea, shallow water, intertidal zone, and land. Furthermore, the genomic resources also provide powerful tools for broader studies on the ecology, evolutionary biology, and biological conservation of isopods.

## Methods

### Sampling and sequencing

The specimens of *B. jamesi* were collected by a deep-sea lander at a depth of 898 m near Hainan Island, in the northern South China Sea (17° 46.845′ N, 110° 38.217′ E). The specimens were identified as the species *B. jamesi* and kept in 75% ethanol and − 80 °C freezer [24]. The muscle of the legs of *B. jamesi* was collected for DNA extraction and genome sequencing. Total genomic DNA was extracted using TIANamp Marine Animal DNA Kits (Tiangen, Beijing, China) and used for Illumina and PacBio sequencing.

For Illumina sequencing, paired-end libraries with short insert size (350 bp) were constructed according to the instructions of the Illumina library preparation kit (Illumina, San Diego, USA). The constructed libraries were sequenced on an Illumina HiseqX-ten sequencing platform (Illumina, San Diego, USA). The raw sequencing reads were trimmed for quality subsequently using Trimmomatic v.0.35 (http://www.usadellab.org/cms/index.php?page=trimmomatic), and the retained clean reads were used for subsequent analyses.

For PacBio sequencing, genomic DNA was sheared to ~ 20 Kb, and the short fragments below the size of 10 Kb were filtered out using BluePippin (Sage Science, Beverly, USA). Filtered DNA was then used for the construction of the proprietary SMRTbell library using PacBio DNA Template Preparation Kit. SMRTbell libraries were used for single-molecule real time (SMRT) sequencing using the P6C5 sequencing chemistry (Pacific Biosciences, San Diego, USA), and then sequenced on the PacBio RSII sequencing platform (Pacific Biosciences, San Diego, USA).

### RNA extraction and sequencing

In order to perform gene annotation and identification of tissue-specific expression genes, transcriptome sequencing was performed on six tissues of *B. jamesi*, namely gill, hepatopancreas, muscle, stomach, intestine, and nerve. According to the standard manufacturer’s protocol, total RNA was isolated and purified from each tissue using TRIzol extraction reagent (Thermo Fisher Scientific, Waltham, USA). RNA quality was determined by 1% agarose gel electrophoresis, and RNA concentration was assessed using a Nanodrop 2000 spectrophotometer (Thermo Fisher Scientific, Waltham, USA). Transcriptome libraries were prepared according to the instructions of the TruSeq RNA Library Prep Kit (Illumina, San Diego, USA), and then sequenced on the Illumina HiSeq 2500 platform. The transcriptome reads were mapped to the genome using TopHat v1.2.1 [56]. Then, fragments per kilobase of transcript per million fragments mapped (FPKM) was calculated using Cufflinks v2.2.1 (http://cole-trapnell-lab.github.io/cufflinks/). The differential gene expression analysis was conducted using edgeR V3.10 [57].

### Genome size estimation

Genome size of *B. jamesi* was estimated by K-mer analysis, which is widely used for the estimation of genome size and repeat content. Jellyfish was used to calculate K-mer frequencies based on the high-quality reads from the Illumina sequencing data [58]. A K-mer depth distribution was plotted and the peak depth could be identified. The genome size was estimated as the ratio of the total number of K-mers to the peak depth.

### Genome assembly and quality assessment

The *B. jamesi* genome was *de novo* assembled based on PacBio subreads using FALCON pipeline (https://github.com/PacificBiosciences/FALCON/) with default parameters. The assembled sequences were then polished using Quiver (SMRT Analysis v2.3.0) based on the alignments of PacBio reads to the assembly. Besides, in order to make the genome assembly more accurate, five rounds of iterative error correction were performed using the aforementioned Illumina clean data.

To assess the quality of the genome assembly, Illumina sequencing reads were aligned to the genome using Bowtie2 and the genome coverage was calculated [59]. Besides, the unigenes obtained from the transcriptome data were mapped to the *B. jamesi* genome to assess the completeness of the gene regions. In addition, the sets of Benchmarking universal single-copy orthologs (BUSCO) was used to evaluate the completeness of the genome assembly (http://gitlab.com/ezlab/busco).

### Repetitive sequence annotation

TEs in the *B. jamesi* genome were predicted by a combination of de novo-based and homology-based approaches. For TE annotation, both RepeatModeler and RepeatMasker were used to perform *de novo* identification [60]. RepeatMasker was used to identify transposable elements by aligning the genome assembly against the RepBase (RepBase21.04) and a local library generated by RepeatModeler with default parameters.

For phylogenetic analysis of TEs, MUSCLE v5 was used for generating multiple alignments of each cluster of TEs in a fast mode (-maxiters 2 -diags1) [61]. Based on the alignment results, the maximum likelihood (ML) method was used for phylogenetic tree construction with the parameters of “-n 1 -o tl -m 012345.” The visualization of the tree was performed on the iTOL (https://itol.embl.de/).

### Protein-coding gene prediction and annotation

Protein-coding genes were predicted through the combination of *de novo* prediction, homology-based prediction and transcriptome-based prediction methods. For *de novo* prediction, the coding regions of the repeat-masked genome were predicted by Augustus v2.5.5 [62]. For homology-based prediction, protein-coding genes from *Daphnia pulex*, *E. texana*, *Litopenaeus vannamei*, *Parhyale hawaiensis*, *Drosophila melanogaster*, *Bombyx mori*, and *Anopheles gambiae* were downloaded from NCBI and mapped against the *B. jamesi* genome with Exonerate v2.2.0 (http://www.ebi.ac.uk/~guy/exonerate/). For transcriptome-based prediction, the transcriptome data was aligned to the *B. jamesi* genome using Tophat v2.1.1. Then, Cufflinks v2.2.1 was used to convert the transcripts to gene models [56]. Finally, all gene models predicted by above three methods were integrated into a non-redundant gene set through EvidenceModeler (EVM) v1.1.1 [63].

Functional annotation of the predicted genes was conducted by blasting against the NR and SwissProt databases using BLASTP program. Protein domains were annotated by mapping the genome to the InterPro and Pfam databases using InterProScan v5.50 and HMMER v3.3.1 [64, 65]. KEGG Automatic Annotation Server (KAAS) was used to annotate the pathways in which the genes might be involved through mapping against the KEGG database (https://www.genome.jp/kaas-bin/kaas_main). The GO classifications of the genes were extracted from the corresponding InterProScan or Pfam results (http://geneontology.org/docs/go-annotations/).

### Gene family analyses

To understand the evolutionary dynamics of the genes, gene family clustering analysis was performed using the Markov clustering program OrthoFinder [66]. An all-to-all blast search was conducted on the protein-coding genes of 11 arthropods, including *B. jamesi*, *A. vulgare*, *D. pulex*, *E. texana*, *Eurytemora affinis*, *L. vannamei*, *Eriocheir sinensis*, *Procambarus virginalis*, *P. hawaiensis*, *Tigriopus californicus*, and *D. melanogaster*, using BLASTP program with a threshold value of E ≤ 1E−05.

Expansion and contraction of the gene families among these 11 species were determined. Based on the clustering results calculated by OrthoFinder and cladogram of these 11 species, gene gain and loss analysis was conducted by CAFE 5 [67]. The expansion and contraction of each gene family was examined by comparing cluster size differences between the ancestor and each of the current species. A random birth and death process model was used to identify gene gain and loss along each lineage of the RAxML tree.

### Phylogenetic analysis

According to the results of gene family clustering, 177 single-copy orthologous genes were selected for phylogenetic tree construction. For each ortholog group, the amino acid sequence alignment was conducted using MUSCLE v5 with the default settings [61]. The 177 protein alignments were merged to form a super alignment matrix. Then, the ML method was used for phylogenetic tree construction under the PROTGAMMAJTT model using RAxML [68]. ML phylogeny and branch lengths were obtained by RAxML with 1000 bootstrap replicates. The divergence time estimation was conducted by combining programs of r8s and RAxML [69]. Fossil-derived timescales and evolutionary history were obtained from TIMETREE (http://timetree.org/).

### Whole-genome duplication analysis

To infer WGD events in *B. jamesi*, we performed a series of analyses on the *B. jamesi* genome, including intrachromosome synteny block identification, Hox gene cluster comparison, and synonymous substitution (Ks) distribution analysis. To identify the synteny blocks, an all-against-all BLASTP method (E value < 1E−5) was used to detect paralogous genes in the *B. jamesi* genome, as well as the genomes of *A. vulgare*, *Daphnia magna*, *T. tridentatus*, and *L. vannamei*. Synteny blocks with at least five collinear homologous genes were detected using MCScanX software [70] with the following standard parameters: MATCH_SCORE: 50, MATCH_SIZE: 20, GAP_PENALTY: -1, OVERLAP_WINDOW: 5, E_VALUE: 1e-05, and MAX GAPS: 25. Genes were further classified by duplicate gene-classifier in MCScanX. For the Ks distribution analysis, the Ks values of the blocks from intraspecies were calculated using the HKY model [71, 72]. The Hox gene cluster, which contains at least nine highly conserved Hox genes, was identified in seven arthropod genomes, including a species with WGD, *T. tridentatus* [73].

### Positive selection analysis

The nonsynonymous/synonymous substitution ratio (ω = *d*_N_/*d*_S_) was calculated to identify positively selected genes. The alignment of the orthologous genes was conducted with MUSCLE, and the stop codon and gaps in the alignment were removed. PAML was used to predict positively selected genes using the branch model [74, 75]. For the branch model test, positive selection was indicated when a significant difference between the alternative and null models was observed using the likelihood ratio test (LRT). The null model assumes that ω is constant (ω = 1), whereas the alternative model allows ω to vary among different branches.

## Supplementary Information

**Additional file 1: Table S1**. Statistics of genome sequencing data of *B. jamesi*. **Table S2.** Genome size and repeat contents of the sequenced crustacean genomes. **Table S3.** Summary of the Illumina sequencing reads coverage on the assembly genome. **Table S4.** Core gene estimation for *B. jamesi* assembly. **Table S5.** Summary of isopod genome sizes. **Table S6.** Summary of the orthologous gene clusters analyzed in 11 species. **Table S7.** The gene family analysis results using CAFE. **Table S8.** GO enrichment of the expanded gene families of *B. jamesi*. **Figure S1.** K-mer distribution of the *B. jamesi* genome sequences. **Figure S2.** The core gene coverage of crustacean genomes. **Figure S3.** A Venn diagram of the statistics of the functional annotation. **Figure S4.** Whole-genome duplication analysis of *B. jamesi*. **Figure S5.** Age distribution of major expanded TEs in the two isopod genomes. **Figure S6.** The TE distribution surrounding genes. **Figure S7.** KEGG enrichment of the expanded gene families of *B. jamesi*. **Figure S8.** Expression level of the genes involved in the glycolysis of *B. jamesi*. **Figure S9.** The distribution ranges and max body lengths of the species of genus *Bathynomus*.

## Data Availability

All PacBio long-read sequencing data are available in the NCBI SRA database under accession ID of SRR16962112-SRR16962114. The genome assembly is available in the NCBI under Bioproject ID PRJNA776076 [76]. The genome assembly, predicted genes, repeats, and all raw sequencing data of genome and transcriptome are also available on the database at the link (username: sph; password: sph@8786@326): http://210.72.156.40/download/sphaeromadae/. In order to perform Protein-coding gene annotation and comparative genomics analysis, the genomes of 14 arthropods were downloaded from NCBI, including *A. vulgare* (PRJNA501402) [14], *D. pulex* (PRJNA12756) [77], *E. texana* (PRJNA352082) [78], *E. affinis* (PRJNA423276) [79], *L. vannamei* (PRJNA438564) [27], *E. sinensis* (PRJNA238496) [26], *P. virginalis* (PRJNA356499) [80], *P. hawaiensis* (PRJNA306836) [81], *T. californicus* (PRJNA237968) [82], *D. magna* (PRJNA490418) [83], *T. tridentatus* (PRJNA510236) [4], *D. melanogaster* (PRJNA559813) [84, 85], *B. mori* (PRJDB4947) [86], and *A. gambiae* (PRJNA20301) [75].

## Abbreviations

TEs: Transposable elements
SSRs: Simple sequence repeats
LINEs: Long interspersed nuclear elements
LTRs: Long terminal repeats
Mya: Million years ago
GO: Gene ontology
CAMs: Cell adhesion molecules
PLC: Phosphatidylinositol phospholipase C
ITPR1: Inositol 1,4,5-triphosphate receptor type 1
TPK: Tyrosine-protein kinase
ADCY: Adenylate cyclase
MTOR: Serine/threonine-protein kinase mTOR
TSC2: Tuberous sclerosis 2
MED: Mediator of RNA polymerase II transcription subunit
TH: Thyroid hormone
TPO: Thyroid peroxidase
TRα: Thyroid hormone receptor α
TRβ: Thyroid hormone receptor β
TRIP11: Thyroid receptor-interacting protein 11
IGF: Insulin growth factor
IGFBP: Insulin-like growth factor-binding protein
ISL: Insulin enhancer protein
IR: IGF receptor
IGFBP-ALS: Insulin-like growth factor-binding protein complex acid labile subunit
PTPRF: Receptor-type tyrosine-protein phosphatase F
LIPE: Hormone-sensitive lipase
PHKA_B: Phosphorylase kinase alpha/beta subunit
FLOT: Flotillin
HCSH: Dachsous
KIBRA: Protein Kibra
YAP1: Transcriptional coactivator YAP1
PP2A: Serine/threonine-protein phosphatase 2A
SHOX: Short-stature homeobox protein
HMG: High mobility group protein
PGM2: Phosphoglucomutase-2
ACSS1_2: Acetyl-CoA synthetase
ADH: Alcohol dehydrogenase
AnxB9: Annexin B9
BMR: Basal metabolic rate
SMRT: Single-molecule real time
FPKM: Fragments per kilobase of transcript per million fragments mapped
BUSCO: Benchmarking universal single-copy orthologs
ML: Maximum likelihood
KAAS: KEGG Automatic Annotation Server
LRT: Likelihood ratio test
