# Supplementary material for: Genome of a giant isopod, Bathynomus jamesi, provides insights into body size evolution and adaptation to deep-sea environment
Source: BMC Biol. 2022 May 13;20:113. doi: 10.1186/s12915-022-01302-6 (PMC9107163; doi:10.1186/s12915-022-01302-6)
Supplement: Supplementary file 1 — Additional file 1: Table S1. Statistics of genome sequencing data of B. jamesi. Table S2. Genome size and repeat contents of the sequenced crustacean genomes. Table S3. Summary of the Illumina sequencing reads coverage on the assembly genome. Table S4. Core gene estimation for B. jamesi assembly. Table S5. Summary of isopod genome sizes. Table S6. Summary of the orthologous gene clusters analyzed in 11 species. Table S7. The gene family analysis results using CAFE. Table S8. GO enrichment of the expanded gene families of B. jamesi. Figure S1. K-mer distribution of the B. jamesi genome sequences. Figure S2. The core gene coverage of crustacean genomes. Figure S3. A Venn diagram of the statistics of the functional annotation. Figure S4. Whole-genome duplication analysis of B. jamesi. Figure S5. Age distribution of major expanded TEs in the two isopod genomes. Figure S6. The TE distribution surrounding genes. Figure S7. KEGG enrichment of the expanded gene families of B. jamesi. Figure S8. Expression level of the genes involved in the glycolysis of B. jamesi. Figure S9. The distribution ranges and max body lengths of the species of genus Bathynomus. [file 12915_2022_1302_MOESM1_ESM.pdf]

Supplementary Information for

Genome of a giant isopod, *Bathynomus jamesi*, provides insights into body size evolution and adaptation to deep-sea environment

Jianbo Yuan[1,2,#], Xiaojun Zhang[1,2,#], Qi Kou[1,2,#], Yamin Sun[3], Chengzhang Liu[1,2], Shihao Li[1,2], Yang Yu[1,2], Chengsong Zhang[1,2], Songjun Jin[1,2], Jianhai Xiang[1,2,*], Xinzheng Li[1,2,*], Fuhua Li[1,2,*]

[1] CAS and Shandong Province Key Laboratory of Experimental Marine Biology, Department of Marine Organism Taxonomy & Phylogeny, Center for Ocean Mega-Science, Institute of Oceanology, Chinese Academy of Sciences, Qingdao 266071, China.

[2] Laboratory for Marine Biology and Biotechnology, Qingdao National Laboratory for Marine Science and Technology, Qingdao 266237, China.

[3] Research Center for Functional Genomics and Biochip, Tianjin 300457, China.

[#]These authors contributed equally. [*]Correspondence and requests for materials should be addressed to F.L. (fhli@qdio.ac.cn, Orcid ID: 0000-0001-8693-600X), or to X.L. (lixzh@qdio.ac.cn). or to J.X. (jhxiang@qdio.ac.cn, Orcid ID: 0000-0001-5395-7787).

Supplementary tables

Table S1. Statistics of genome sequencing data of *B. jamesi*.

Clean Reads	Illumina	PacBio
Size of library	500 bp	20 Kb
Number of reads	784,169,503 × 2	31,925,544
Average length of reads	150 bp	12,983 bp
Total bases	235.25 Gb	360.80 Gb
Sequencing depth *	~45X	~69X

* Genome size ~ 5.24 Gb.

Table S2. Genome size and repeat contents of the sequenced crustacean genomes.

Species	Class	Genome size (Gb)	Repeat contents
Armadillidium vulgare	Isopoda	1.73	69.54%
Bathynomus jamesi	Isopoda	5.90	85.24%
Litopenaeus vannamei	Decapoda	1.66	49.39%
Fenneropenaeus chinensis	Decapoda	1.57	49.71%
Marsupenaeus japonicus	Decapoda	1.79	34.96%
Penaeus monodon	Decapoda	2.39	62.50%
Procambarus virginalis	Decapoda	3.29	26.59%
Macrobrachium nipponense	Decapoda	4.49	50.20%
Scylla paramamosain	Decapoda	1.55	35.79%
Eriocheir sinensis	Decapoda	1.56	35.57%
Paralithodes platypus	Decapoda	4.80	77.73%
Portunus trituberculatus	Decapoda	1.00	54.52%
Callinectes sapidus	Decapoda	0.99	36.00%
Procambarus fallax	Decapoda	3.30	8.80%
Homarus americanus	Decapoda	2.29	52.90%
Daphnia pulex	Branchiopoda	0.20	20.45%
Eulimnadia texana	Branchiopoda	0.12	26.40%
Artemia franciscana	Branchiopoda	0.85	58.00%
Tigriopus californicus	Copepoda	0.24	39.00%
Parhyale hawaiensis	Amphipoda	4.02	57.00%
Trinorchestia longiramus	Amphipoda	0.89	20.35%
Amphibalanus amphitrite	Cirripedia	0.48	4.48%
Lepas anserifera	Cirripedia	0.75	44.70%

Table S3. Summary of the Illumina sequencing reads coverage on the assembly genome.

**Coverage of Illumina sequencing reads**	
Number of clean reads	1,520,127,222
Percentage of mapped reads	99.80%
Coverage	96.11%
Coverage at least 5X	90.88%
Coverage at least 10X	85.12%
Coverage at least 20X	71.36%
Coverage at least 50X	21.30%
**Coverage of RNA-seq sequencing reads**	
Number of clean reads	313,907,086
Percentage of mapped reads	84.23%

Table S4. Core gene estimation for *B. jamesi* assembly.

	**Number**	**Percentage (%)**
**Complete BUSCOs**	248	81.9
**Complete and single-copy BUSCOs**	219	72.3
**Complete and duplicated BUSCOs**	29	9.6
**Fragmented BUSCOs**	39	12.9
**Missing BUSCOs**	16	5.2
**Total BUSCO groups searched**	303	100

Table S5. Summary of isopod genome sizes.*

Family	Species	Common Name	Inhabit	C-value
Armadillidiidae	*Armadillidium sp.*	Pill bug	Terrestrial	2.35
Armadillidiidae	*Armadillidium vulgare*	Pill bug	Terrestrial	1.96
Asellidae	*Caecidotea forbesi*	Freshwater isopod	Freshwater	1.90
Asellidae	*Caecidotea racovitzae*	Freshwater isopod	Freshwater	1.71
Cirolanidae	Unknown sp.	Marine isopod	Unknown	2.40
Cymothoidae	*Nerocila munda*	Parasitic isopod	Parasitic	8.82
Unknown	Unknown sp.	Terrestrial isopod	Terrestrial	5.70
Ligiidae	*Ligia hawaiensis*	Sea roach	Intertidal	3.00
Ligiidae	*Ligia occidentalis*	Western sea roach	Intertidal	3.30
Oniscidae	*Oniscus asellus*	Sow bug	Terrestrial	8.60
Porcellionidae	*Porcellio scaber*	Sow bug	Terrestrial	5.65
Porcellionidae	*Porcellio spinicornis*	Sow bug	Terrestrial	3.56
Sphaeromatidae	*Sphaeroma destructor*	Marine isopod	Shallow	6.79
Sphaeromatidae	Unknown sp.	Marine isopod	Unknown	2.80

* The C-values of isopods are referred to the Animal Genome Size Database (www.genomesize.com).

Table S6. Summary of the orthologous gene clusters analyzed in 11 species.

Species name	No. of coding genes	No. of gene families	No. of specific gene families
Bathynomus jamesi	23,221	9,268	3,683
Armadillidium vulgare	19,051	7,664	5,294
Daphnia pulex	30,595	7,894	14,264
Drosophila melanogaster	13,916	6,497	4,403
Eriocheir sinensis	28,033	8,288	9,515
Eulimnadia texana	23,965	7,747	6,087
Eurytemora affinis	19,856	7,733	6,882
Litopenaeus vannamei	28,273	8,660	9,543
Parhyale hawaiensis	28,666	8,983	11,150
Procambarus virginalis	21,773	6,100	11,036
Tigriopus californicus	14,536	7,460	3,099

Table S7. The gene family analysis results using CAFE.

Species	Expanded families	Significantly expanded (*P* < 0.05)	Contracted families	Significantly contracted (*P* < 0.05)
Bathynomus jamesi	2,428	274	814	157
Armadillidium vulgare	907	133	1,896	279
Daphnia pulex	1,130	227	1,534	260
Drosophila melanogaster	640	85	3,186	488
Eriocheir sinensis	1,687	370	1,966	199
Eulimnadia texana	2,650	260	1,358	208
Eurytemora affinis	1,219	131	1,158	235
Litopenaeus vannamei	1,459	270	749	122
Parhyale hawaiensis	1,352	275	1,838	241
Procambarus virginalis	353	59	2,720	380
Tigriopus californicus	745	111	1,463	267

Table S8. GO enrichment of the expanded gene families of *B. jamesi*.

GO terms	Description	Annotated	Significant	*p*-value
Cellular Component				
GO:0005742	mitochondrial outer membrane translocase complex	13	21	7.53E-08
GO:0016020	membrane	241	2468	7.84E-08
GO:0031968	organelle outer membrane	15	41	7.00E-06
GO:0031967	organelle envelope	28	136	3.10E-05
GO:0031985	Golgi cisterna	6	9	3.06E-04
Molecular Function				
GO:0005544	calcium-dependent phospholipid binding	42	55	4.94E-30
GO:0003950	NAD+ ADP-ribosyltransferase activity	35	48	6.49E-24
GO:0005543	phospholipid binding	42	75	3.63E-22
GO:0008236	serine-type peptidase activity	98	359	1.44E-21
GO:0008233	peptidase activity	139	748	2.06E-14
GO:0008289	lipid binding	43	117	2.27E-14
GO:0004175	endopeptidase activity	99	458	2.27E-14
GO:0070011	peptidase activity, acting on L-amino acid peptides	136	729	2.35E-14
GO:0005509	calcium ion binding	81	371	5.65E-12
GO:0004476	mannose-6-phosphate isomerase activity	10	11	1.27E-08
GO:0046872	metal ion binding	191	1361	3.58E-08
GO:0043169	cation binding	191	1368	5.14E-08
GO:0004197	cysteine-type endopeptidase activity	19	48	4.05E-07
GO:0035257	nuclear hormone receptor binding	8	11	1.08E-05
GO:0042974	retinoic acid receptor binding	8	11	1.08E-05
GO:0051427	hormone receptor binding	8	11	1.08E-05
GO:0016861	intramolecular oxidoreductase activity, interconverting aldoses and ketoses	10	20	5.19E-05
GO:0004143	diacylglycerol kinase activity	7	11	1.89E-04
GO:0004497	monooxygenase activity	14	42	2.00E-04
GO:0016860	intramolecular oxidoreductase activity	10	24	3.48E-04
GO:0008376	acetylgalactosaminyltransferase activity	6	9	5.06E-04
GO:0004180	carboxypeptidase activity	16	60	1.02E-03
Biological Process				
GO:0006508	proteolysis	134	756	1.11E-14
GO:0006801	superoxide metabolic process	14	19	2.89E-09
GO:0072593	reactive oxygen species metabolic process	14	22	4.14E-08
GO:0050790	regulation of catalytic activity	20	74	3.58E-04
GO:0065009	regulation of molecular function	20	74	3.58E-04
GO:0007205	protein kinase C-activating G-protein coupled receptor signaling pathway	7	11	9.36E-04
GO:0007219	Notch signaling pathway	5	7	8.18E-03

Supplementary figures

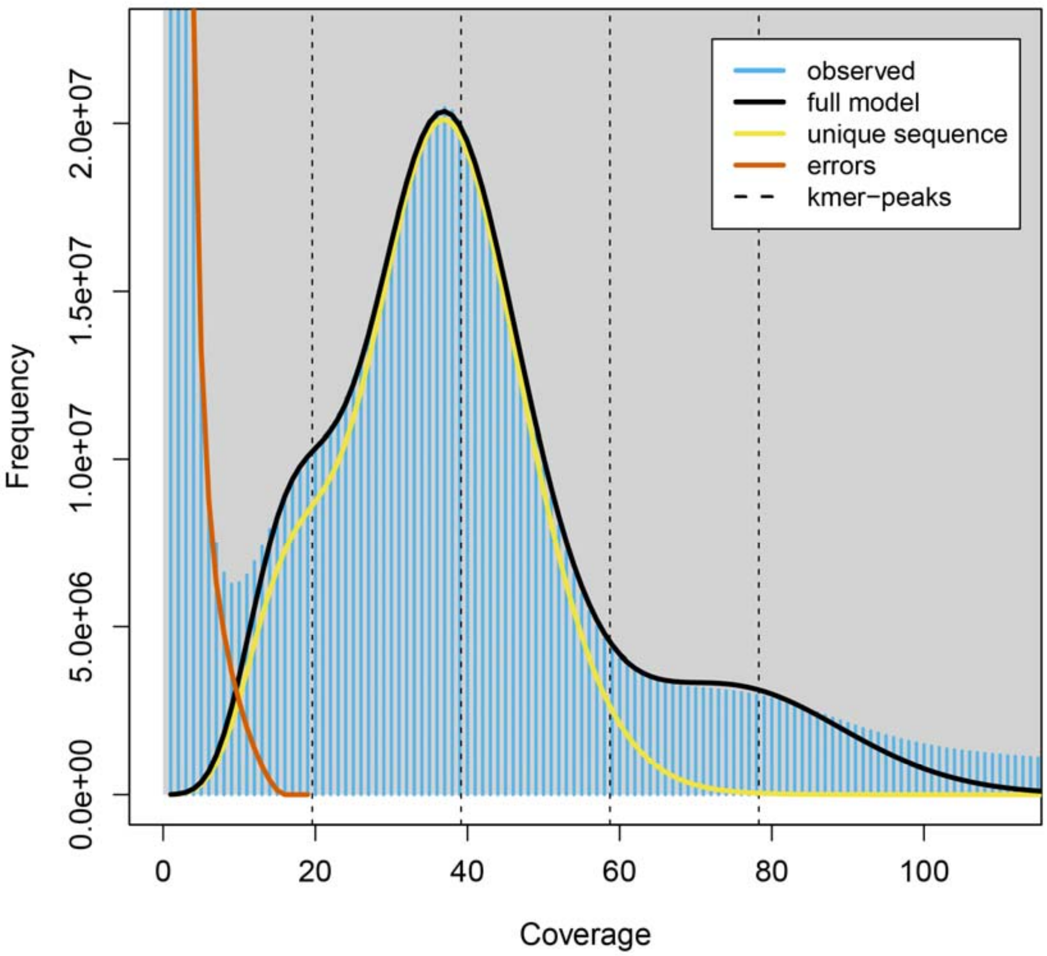

Figure S1. **K-mer distribution of the *B. jamesi* genome sequences.**

K-mer analysis estimated the genome size of *B. jamesi* to be 5.24 Gb. The heterozygous rate was estimated to be 0.69% and the content of repetitive sequences was estimated to be 89.7%.

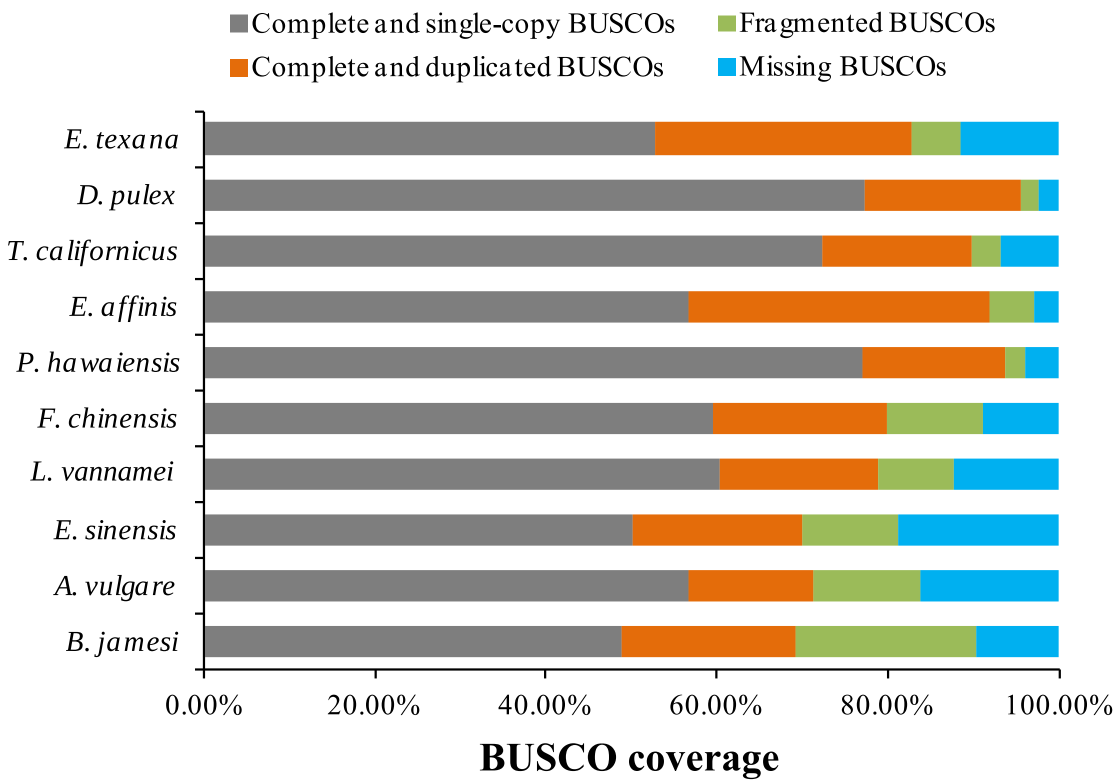

Figure S2. The core gene coverage of crustacean genomes.

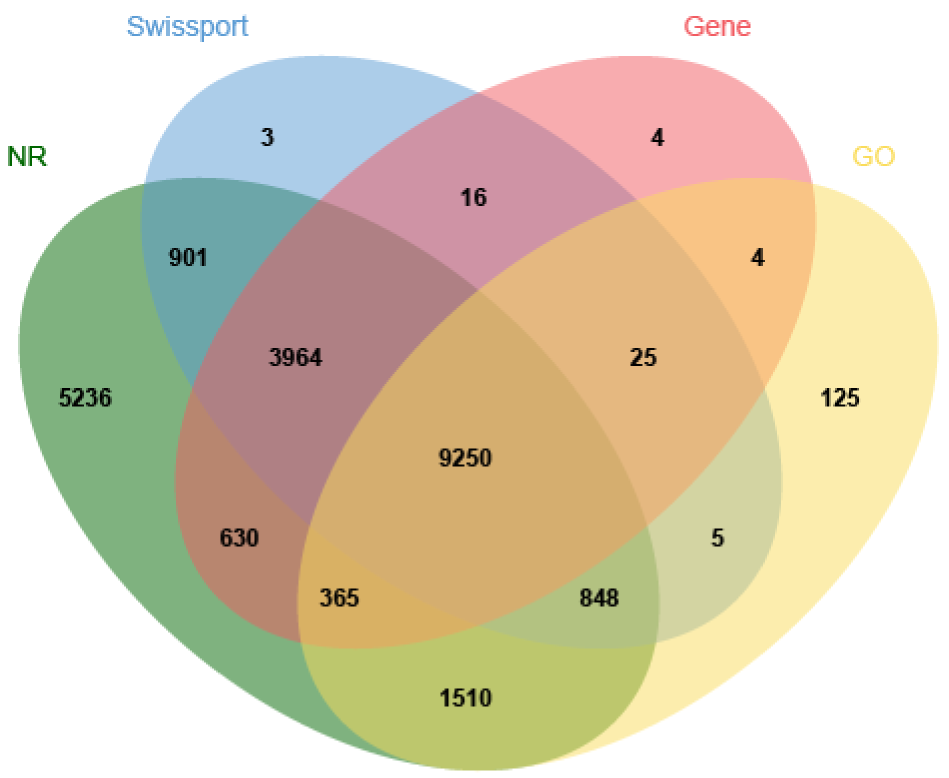

Figure S3. A Venn diagram of the statistics of the functional annotation.

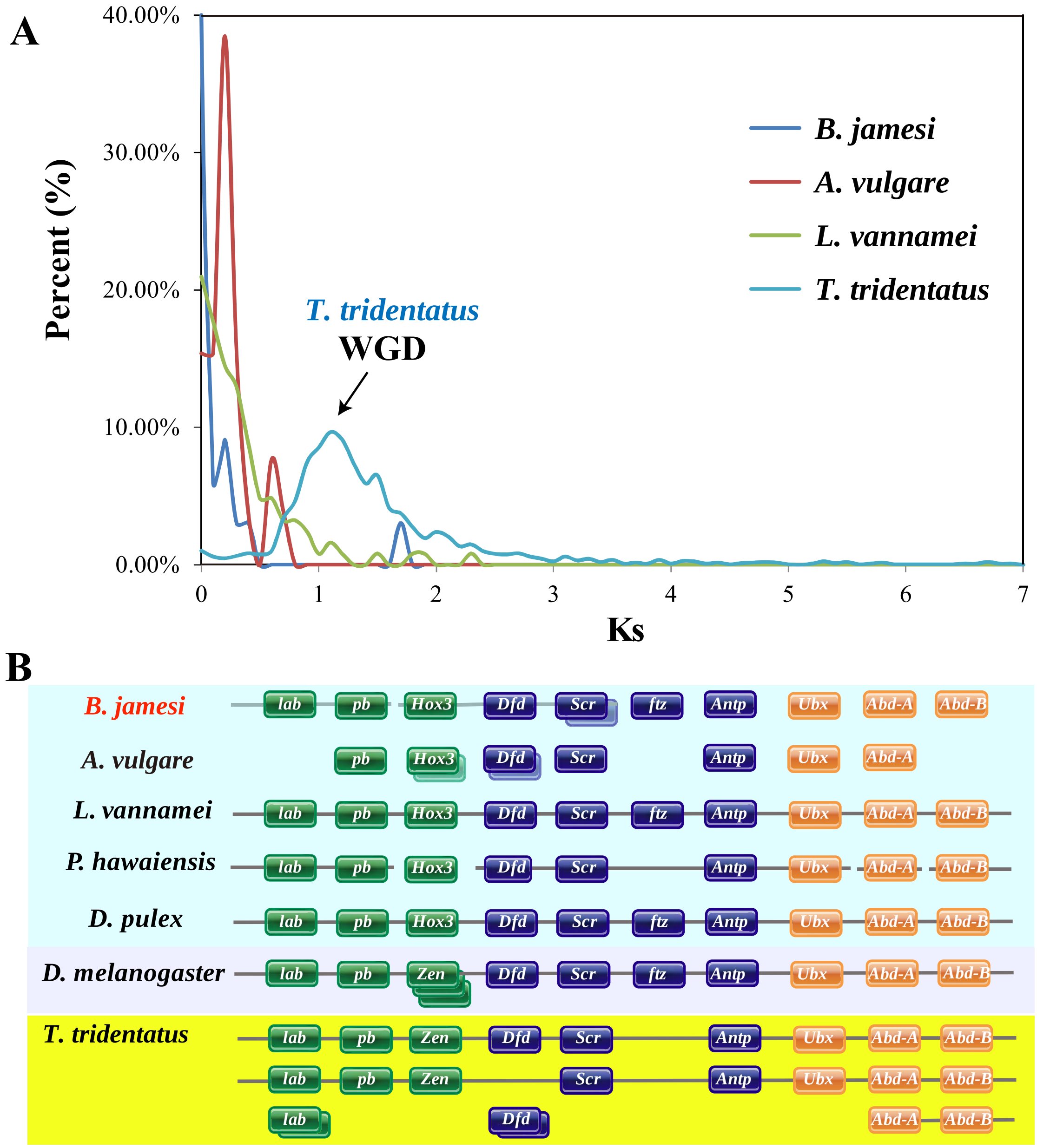

Figure S4. Whole-genome duplication analysis of *B. jamesi*. (A) Distribution of the synonymous substitution rates (Ks) of homologous gene groups for intraspecies comparison. The peak of the arrow indicates WGD event in the *Tachypleus tridentatus* genome. B. jamesi seems have not undergone WGD as no peak relates to WGD has been found in this genome. (**B**) Hox gene clusters of seven arthropods. The boxes connected by a straight line represented ordered genes on a single scaffold or pseudochromosome. Unlike the duplicated Hox gene cluster in the *T. tridentatus* genome, only a single Hox gene cluster identified in both *B. jamesi* and *A. vulgare*.

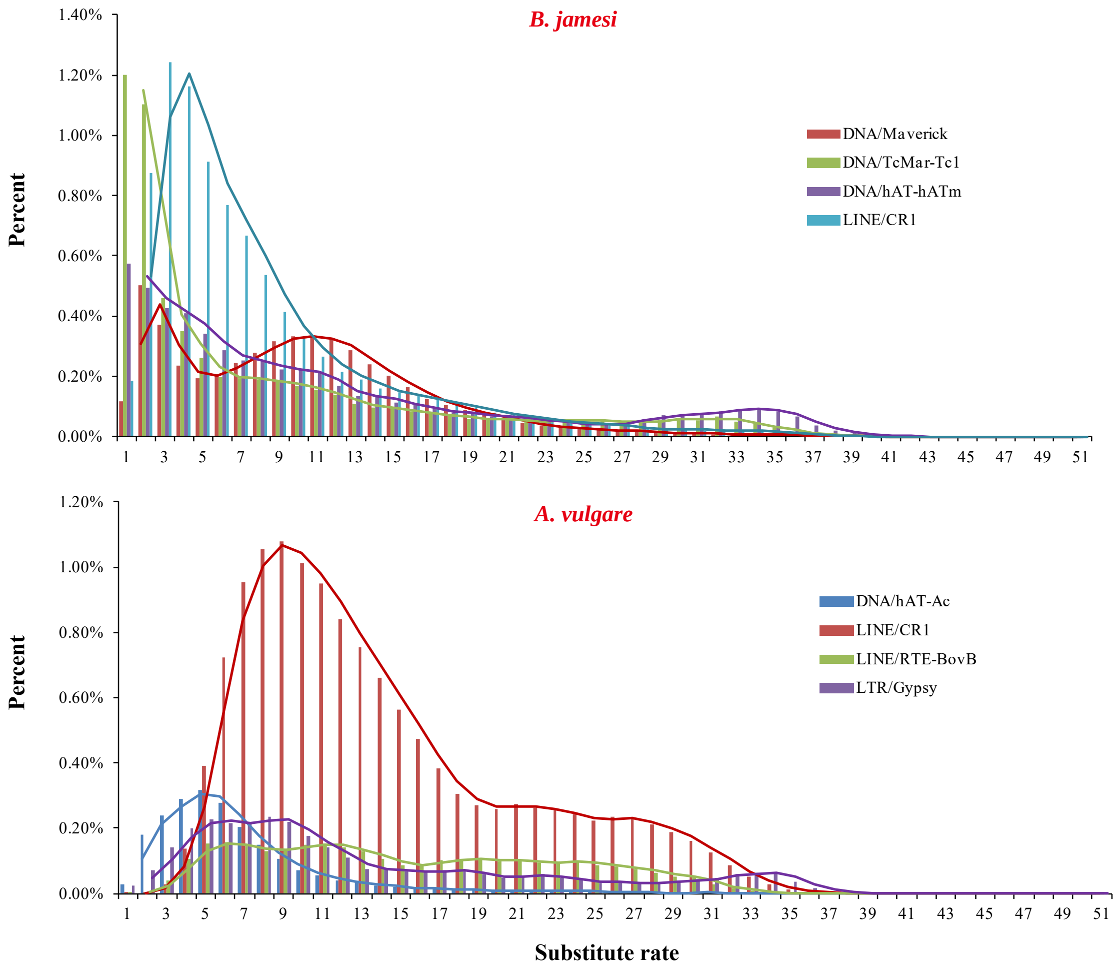

Figure S5. Age distribution of major expanded TEs in the two isopod genomes. The substitution rates were calculated between the genome and repeat consensus sequences.

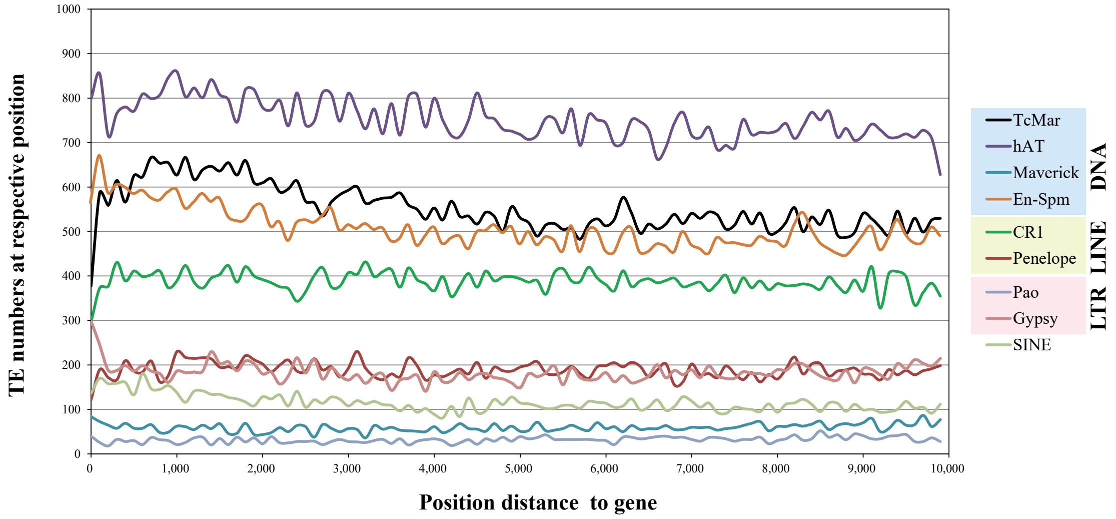

Figure S6. The TE distribution surrounding genes. For each gene, the 10 Kb upstream or downstream of the gene body were analyzed. The plots include only those proliferated in the *B. jamesi* genome.

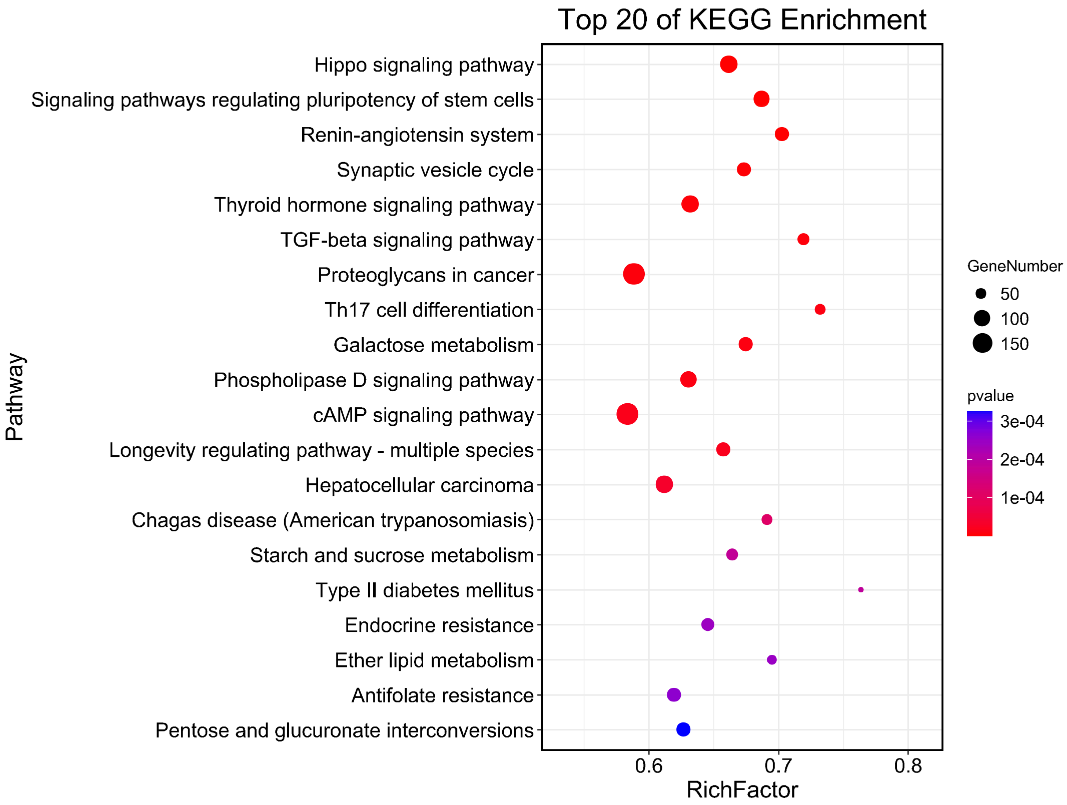

Figure S7. KEGG enrichment of the expanded gene families of *B. jamesi*.

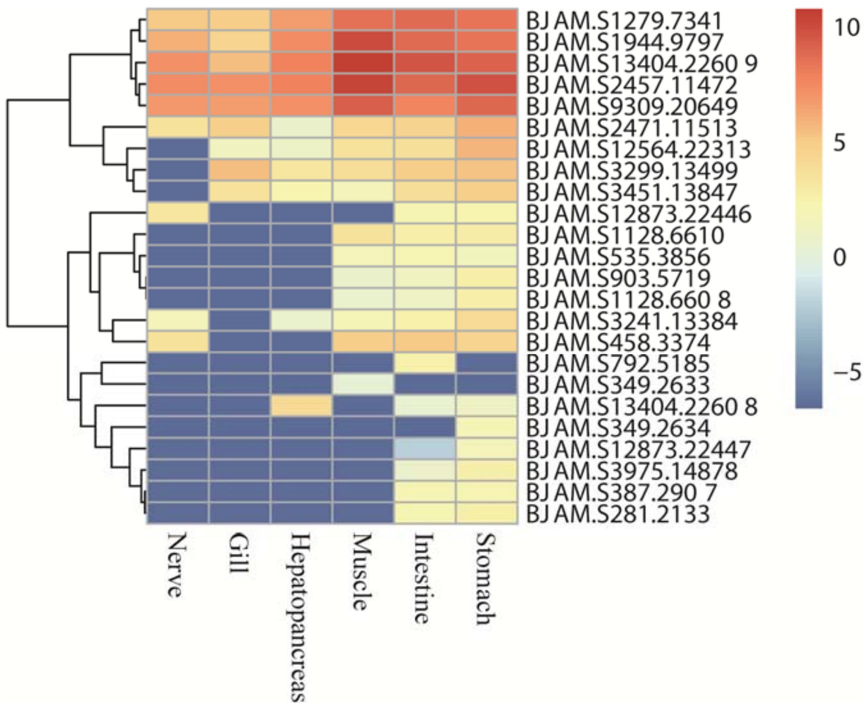

Figure S8. Expression level of the genes involved in the glycolysis of *B. jamesi*.

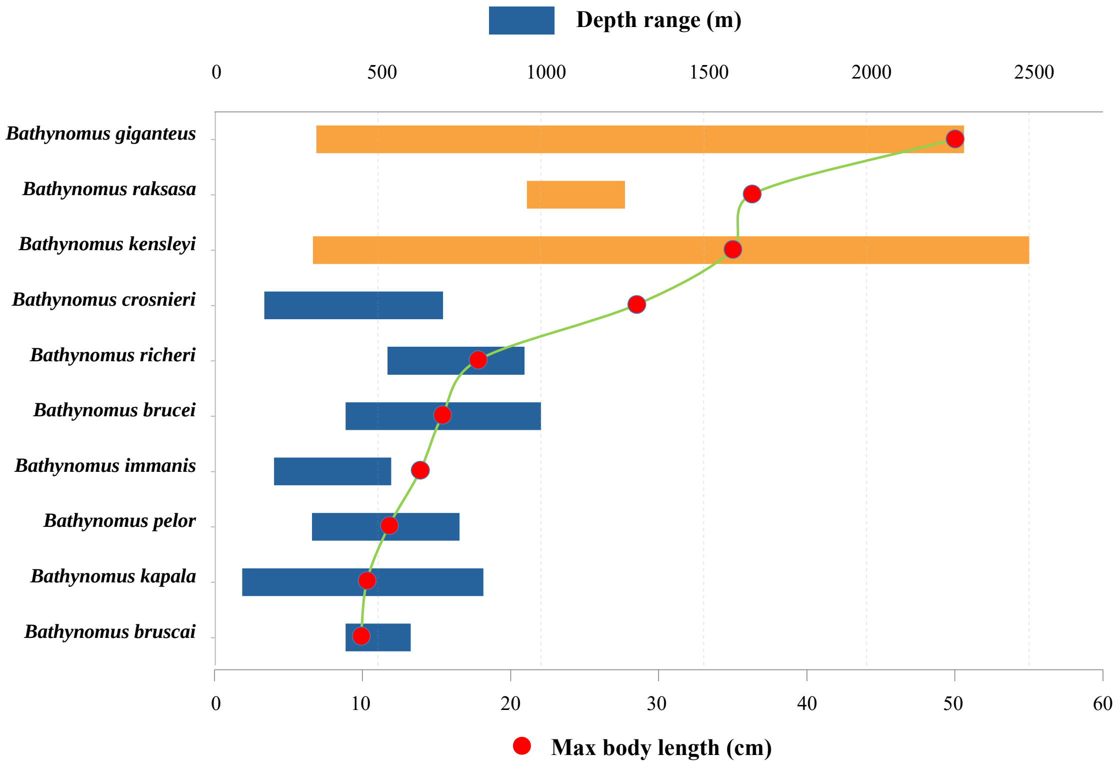

Figure S9. The distribution ranges and max body lengths of the species of genus *Bathynomus*. The information of distribution range and max body length of these species were collected from the SeaLifeBase (https://www.sealifebase.ca/search.php).
